# Identification and Validation of IFI44 as Key Biomarker in Lupus Nephritis

**DOI:** 10.3389/fmed.2021.762848

**Published:** 2021-10-25

**Authors:** Lingling Shen, Lan Lan, Tingting Zhu, Hongjun Chen, Haifeng Gu, Cuili Wang, Ying Chen, Minmin Wang, Haiyan Tu, Philipp Enghard, Hong Jiang, Jianghua Chen

**Affiliations:** ^1^Kidney Disease Center, The First Affiliated Hospital, College of Medicine, Zhejiang University, Hangzhou, China; ^2^Key Laboratory of Nephropathy, Hangzhou, China; ^3^Institute of Nephropathy, Zhejiang University, Hangzhou, China; ^4^Zhejiang Clinical Research Center of Kidney and Urinary System Disease, Hangzhou, China; ^5^Department of Geriatrics, The First Affiliated Hospital, College of Medicine, Zhejiang University, Hangzhou, China; ^6^Department of Nephrology, Zhejiang Provincial People's Hospital, Affiliated People's Hospital, Hangzhou Medical College, Hangzhou, China; ^7^Department of Nephrology, The Second Affiliated Hospital, Zhejiang University School of Medicine, Hangzhou, China; ^8^Department of Nephrology and Medical Intensive Care, Charité - Universitätsmedizin Berlin, Berlin, Germany

**Keywords:** lupus nephritis, weighted gene co-expression network analysis, hub genes, biomarker, type-I interferon

## Abstract

Lupus nephritis (LN) is a common and severe organ manifestation of systemic lupus erythematosus (SLE) and is a major cause of SLE related deaths. Early diagnosis is essential to improve the prognosis of patients with LN. To screen the potential biomarkers associated with LN, we downloaded the gene expression profile of GSE99967 from the Gene Expression Omnibus (GEO) database. Weighted gene co-expression network analysis (WGCNA) was utilized to construct a gene co-expression network and identify gene modules associated with LN. Gene Ontology (GO) analysis was also applied to explore the biological function of genes and identify the key module. Differentially expressed genes (DEGs) were identified and Maximal Clique Centrality (MCC) values were calculated to screen hub genes. Furthermore, we selected promising biomarkers for real-time PCR (qRT-PCR) and enzyme-linked immunosorbent assay (ELISA) validation in independent cohorts. Our results indicated that five hub genes, including IFI44, IFIT3, HERC5, RSAD2, and DDX60 play vital roles in the pathogenesis of LN. Importantly, IFI44 may considered as a key biomarker in LN for its diagnostic capabilities, which is also a promising therapeutic target in the future.

## Introduction

Systemic lupus erythematosus (SLE) is an autoimmune disease involving an inappropriate immune response to endogenous nuclear particles, which affects multiple organs and systems (1). Lupus nephritis (LN) is an immune complex glomerulonephritis that develops as one of the most common and severe target-organ manifestations of SLE (2). The treatment of LN is mainly based on glucocorticoids and immunosuppressants, but the effect is not live up to expectations. Approximately 10–30% of LN patients progress to end-stage renal disease (ESRD) within 15 years after diagnosis, which is the major cause of mortality in SLE (3). Thus, it is imperative to further study the pathogenesis of LN to contribute to the diagnosis and treatment of LN and improve the prognosis of patients with LN.

The pathological mechanism of LN is complicated. Recent studies have shown that LN susceptibility genes, which break immune tolerance are involved in the pathogenesis of LN (4, 5). These genes can enhance the innate immune signaling pathways and promote lymphocytes activation, thus leading to renal damage (4). In addition, autoreactive leukocytes, immune complexes, and complement proteins also play essential roles in LN pathogenesis through various inflammatory mediators (6). However, despite the increased understanding of LN, the genetic and pathogenetic basis of LN remain unclear.

With the rapid development of bioinformatics, microarray data based on high-throughput sequencing has been used to explore the mechanism of diseases at the gene level and identify biomarkers to diagnose diseases or assess prognosis. Weighted gene co-expression network analysis (WGCNA) is a systematics biology method used for integrating gene expression and clinical traits to identify candidate biomarkers and therapeutic targets (7). WGCNA has been widely applied to study various diseases, including cancer (8), autoimmune disease (9), and chronic kidney disease (10). However, there are few studies of WGCNA in LN.

In this study, WGCNA was utilized to construct a gene co-expression network and identify gene modules associated with LN. Gene Ontology (GO) analysis was also applied to explore the potential biological function of genes and identify the key module. Then, we screened five hub genes and verified them by quantitative real-time PCR (qRT-PCR). Furthermore, two promising biomarkers including IFI44 and IFIT3 were selected for enzyme-linked immunosorbent assay (ELISA) validation in an independent cohort. Our findings provide a key biomarker associated with LN pathogenesis and progression, which is helpful for the early diagnosis and treatment of patients with LN.

## Materials and Methods

### Data Collection

**Figure 1:**
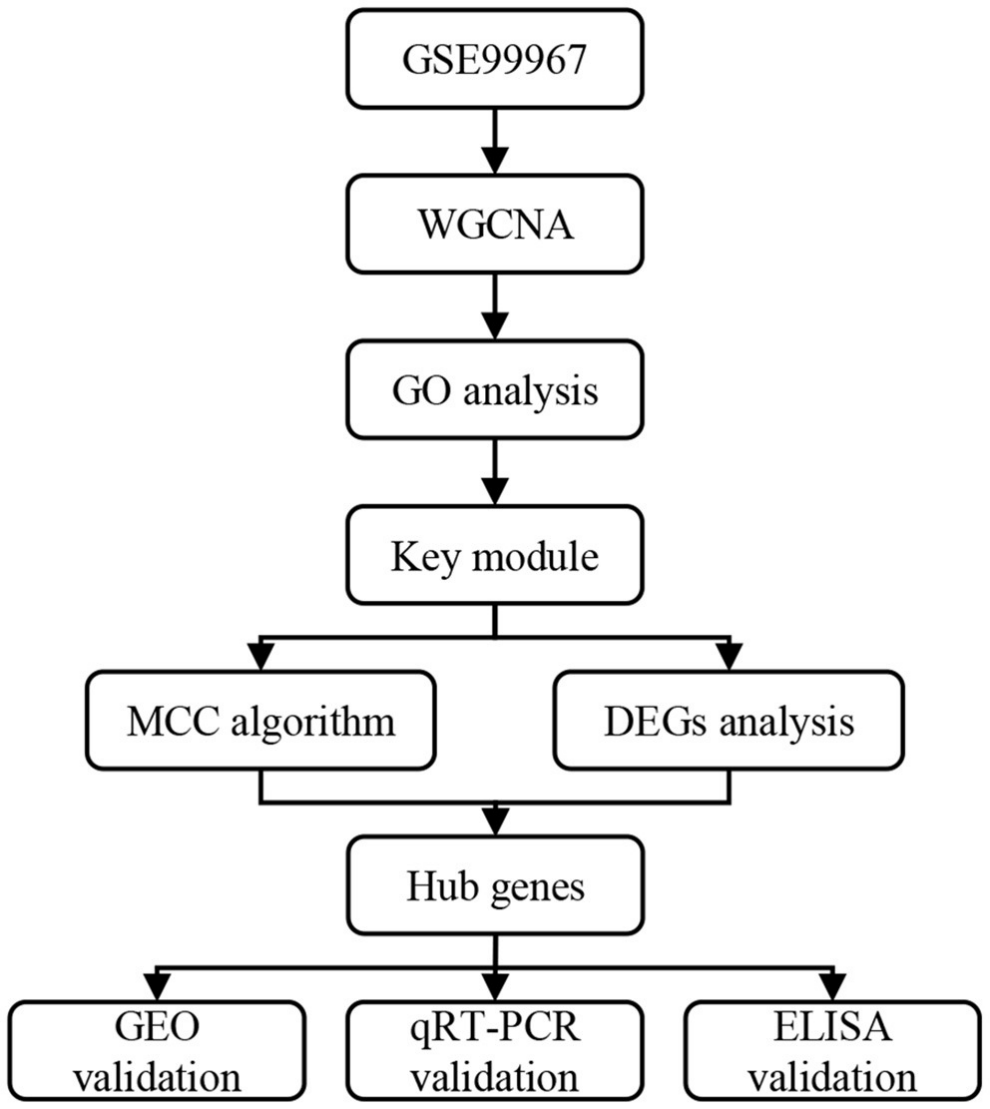
Overall study design. WGCNA, weighted gene co-expression network analysis; GO, gene ontology; MCC, maximal clique centrality; DEGs, differentially expressed genes; GEO, gene expression omnibus; qRT-PCR, quantitative real-time PCR; ELISA, enzyme-linked immunosorbent assay.

Figure 1 shows the overall study design. We downloaded series matrix files of GSE99967 from the Gene Expression Omnibus (GEO) (https://www.ncbi.nlm.nih.gov/geo) database to identify the hub genes related to LN. The platform of the GSE99967 dataset was GPL21970 (Affymetrix Human Gene 2.0 ST Array). After obtaining the data, one sample GSM2666765 was removed for its inconsistent expression data (Supplementary Figure 1). The remaining whole peripheral blood samples from 29 LN patients and 16 normal controls were kept for further analysis (11). The expression data had been log_2_ transformed.

### Weighted Gene Co-expression Network Analysis

The R package “WGCNA” (7) was utilized to construct the co-expression network of all genes in the GSE99967 dataset. Briefly, we calculated Pearson's correlation to construct a pairwise coefficient matrix and then transformed into a weighted adjacency matrix with an appropriate soft-thresholding power. Next, we converted the adjacency matrix into a topological overlap matrix (TOM), and used hierarchical clustering and dynamic tree cut algorithm to classify genes on TOM-based dissimilarity. Then we calculated the correlations of module eigengene (ME), which represents the first principal component of a module, and merged similar modules with highly correlated eigengenes. In addition, a module-trait relationship was calculated based on the correlation between the gene module and clinical trait. Finally, gene significance (GS) which refers to the correlation between gene expression and the clinical trait was used to identify clinical-related modules.

### Functional Enrichment Analysis

To investigate the potential biological function of genes in clinical-related modules, we performed GO enrichment analysis using Database for Annotation, Visualization and Integrated Discovery DAVID (https://david.ncifcrf.gov/). GO terms with *p* < 0.05 were considered statistically significant. The R package “ggplot2” was utilized to show results in the bubble chart.

### Differentially Expressed Gene Analysis

The GEO2R online tools (12) were used to identify differentially expressed genes (DEGs) between LN patients and normal controls. Genes with adjusted *p* < 0.05 and | log_2_ fold change (FC)| ≥ 1 were considered as DEGs. The heatmap and volcano plot of all DEGs were made by the R package “pheatmap” and “ggplot2,” respectively.

### Hub Genes Identification

We used CytoHubba (13), a Cytoscape software (14) plugin to select the hub genes in the key module. The top ten genes with higher maximal clique centrality (MCC) values were screened. Among these ten genes, five DEGs were identified as hub genes. We investigated the GS and module membership (MM) of the selected hub genes to verify their reasonability.

### Validation of Hub Genes in Other Datasets

To validate five hub genes, we downloaded another two datasets GSE72798 (15) and GSE32591 (16) from the GEO database. The dataset GSE72798 consists of 10 LN patients and 10 normal controls and the RNA was extracted from blood samples. In the GSE32591 dataset, the total RNA was extracted from kidney biopsy of 32 LN patients and 14 normal controls.

### Patients and Samples Collection

To further verify selected genes, we recruited 78 LN patients, 67 healthy controls (HCs), and 25 patients with IgA nephropathy from the First Affiliated Hospital, College of Medicine, Zhejiang University. Patients or the public provided written informed consent to participate in this study. All LN patients were confirmed by kidney biopsy, and the Systemic Lupus Erythematosus Disease Activity Index (SLEDAI) was calculated, which contains 24 items reflecting disease activity. Active LN was defined as biopsy-proven active nephritis or SLEDAI ≥10 and at least two renal elements of the SLEDAI; inactive LN was defined as biopsy-proven pure class V or VI nephritis or SLEDAI <10.

Serum was separated from blood samples and stored at −80°C. Peripheral blood mononuclear cells (PBMCs) were isolated by human PBMCs separation medium (tbdscience, Tianjin, China).

### RNA Extraction and qRT-PCR

Total RNA was extracted from PBMCs samples using TRIzol® (Invitrogen, CA, USA) and then reversely transcribed into cDNA by the PrimeScript™ II Reverse Transcriptase (Takara, Shiga, Japan). According to the manufacturer's instructions, reverse transcription was conducted at 37°C for 15 min and 85°C for 5 s. qRT-PCR was performed by SYBR Green on CFX96™ Real-Time PCR Detection Systems (Bio-rad, CA, USA). All primer sequences used were shown in Supplementary Table 1. The conditions of qRT-PCR were as follows: 95°C for 30 s, followed by 40 cycles of 95°C for 5 s and 60°C for 31 s, with dissociation at 95°C for 15 s, 60°C for 1 min and 95°C for 15 s. The average threshold cycle (Ct) was calculated for each transcript, which was performed in triplicate. The relative expression of each mRNA was normalized by GAPDH and analyzed using the 2^−ΔΔCT^ method [ΔΔCT= (CT of gene) – (CT of GAPDH) – (CT of HC)].

### ELISA

The serum levels of IFI44 and IFIT3 were assessed with ELISA kits (G-Biosciences, CA, USA for IFI44, and MyBioSource, CA, USA for IFIT3). Briefly, diluted serum samples were incubated in the microplate. The membrane was then incubated with biotin-labeled antibody after washing twice. Similarly, the membrane was incubated with avidin-labeled horseradish peroxidase (HRP) and then with substrated solution (TMB). Finally, the absorbance values at 450 nm of samples were read, and the concentration of IFI44 and IFIT3 were calculated using standard curves run on each ELISA plate.

### Statistical Analyses

Statistical analyses were performed using SPSS 20 (IBM) and GraphPad Prism 8.0 (GraphPad Software, CA, USA). The unpaired *t*-test was used to analyze differences between two groups. Results were expressed as the mean ± standard deviation (SD). The receiver operator characteristic (ROC) curves were drawn and area under curve (AUC) was calculated to assess the diagnostic capability of biomarkers. Logistic regression analysis was performed to create a combined score. *P* < 0.05 was considered statistically significant.

## Results

### Construction of Weighted Gene Co-expression Network

**Figure 2:**
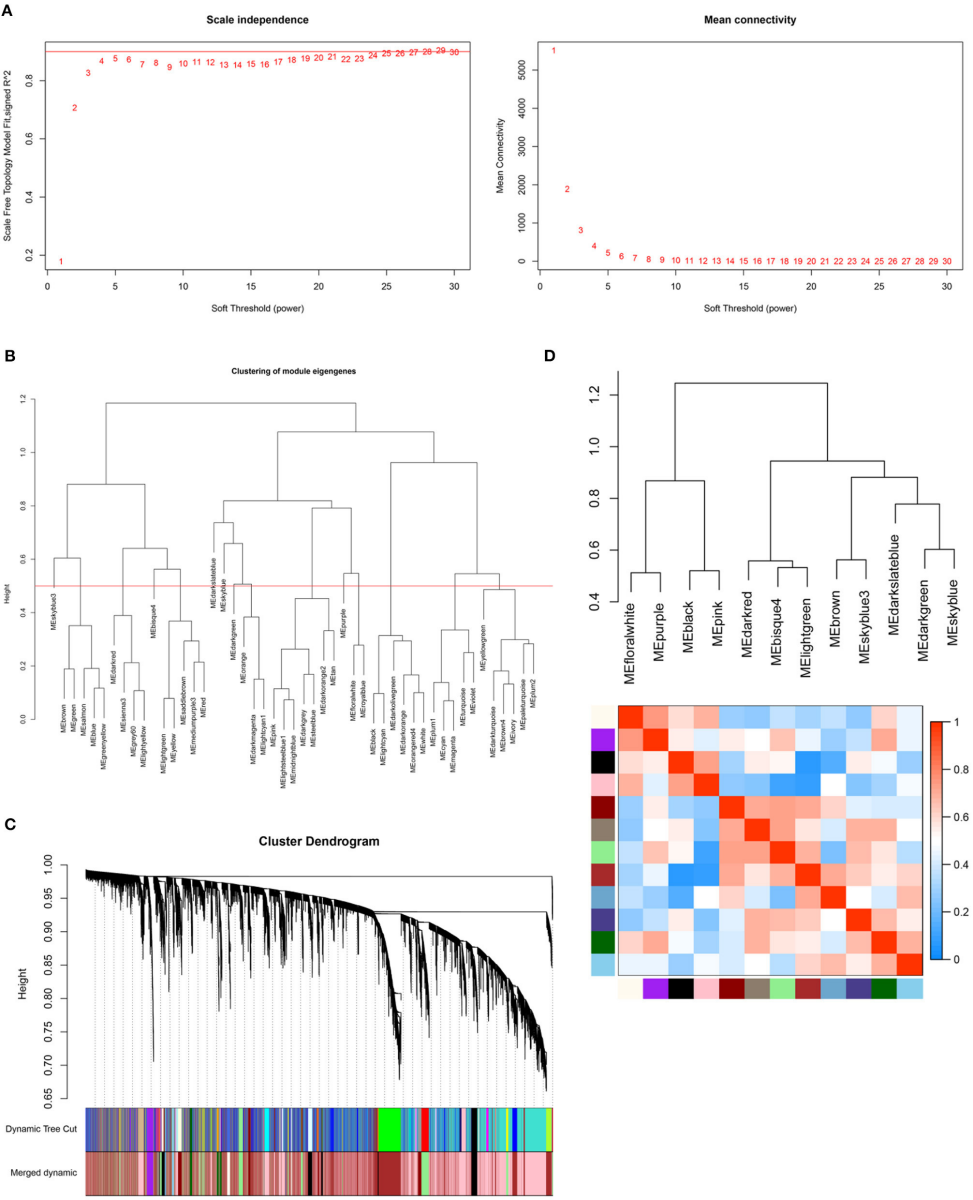
Construction of weighted gene co-expression network. **(A)** Analysis of the scale-free topology model fit index for soft threshold powers (Left) and analysis of the mean connectivity for soft threshold powers (Right). The soft-thresholding power was set as four. **(B)** Clustering of module eigengenes. The red line represents cut height (0.5). **(C)** The cluster dendrogram of genes. **(D)** The cluster dendrogram and adjacency heatmap of eigengenes.

Samples in the GSE99967 dataset were divided into two groups (29 LN samples and 16 normal controls). Expression data of all genes from GSE99967 were used to conduct WGCNA (Figure 2). Based on scale-free topology model fit index and mean connectivity, the soft-threshold power was set as four (Figure 2A). Then, we merged similar modules to acquire 12 co-expression modules by setting the cut height of module eigengenes as 0.5 (Figures 2B,C). Figure 2D showed the cluster dendrogram and adjacency heatmap of eigengenes, which means that 12 modules were primarily separated into two clusters.

### Key Module Identification

**Figure 3:**
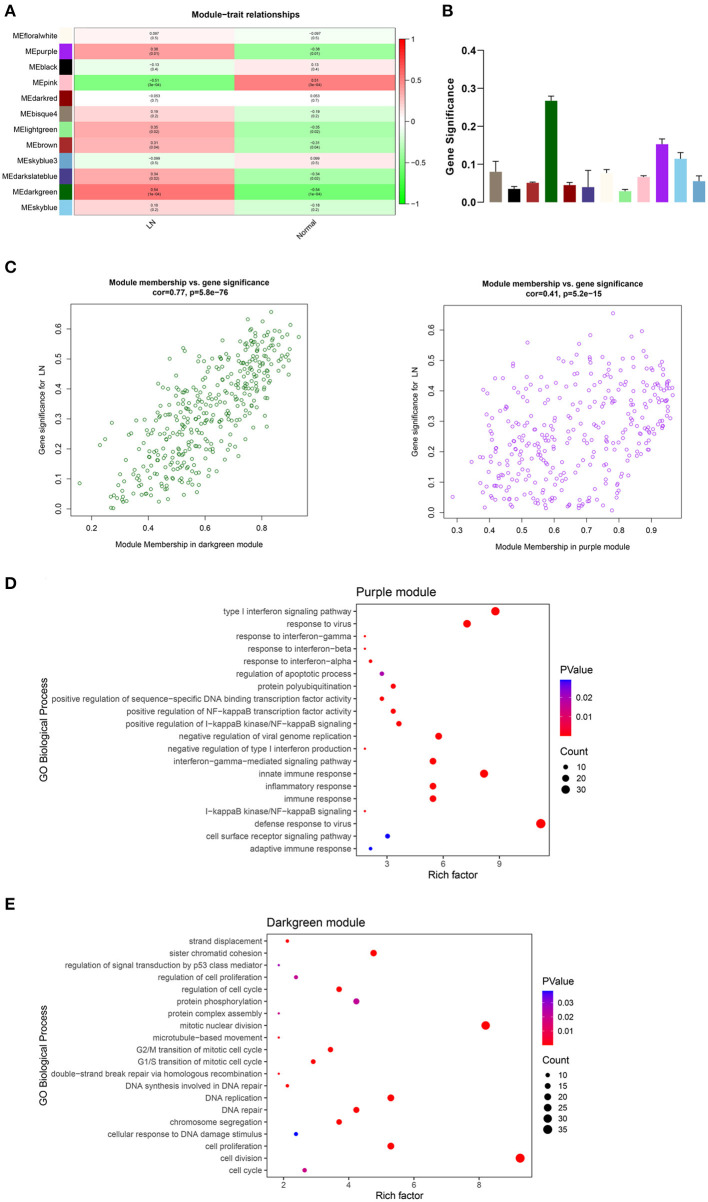
Key module identification. **(A)** Heatmap of module-trait relationships. The correlation coefficients and *p*-value are shown in each cell. Red presents a positive correlation and blue presents a negative correlation. **(B)** Gene significance of all genes in 12 modules related to LN. Different colors of columns present different modules. **(C)** Scatter plot for correlation between module membership and gene significance in darkgreen module (Left) and purple module (Right). The top 20 significant GO terms (biological process) of the purple module **(D)** and darkgreen module **(E)**. The color and size of each point indicate the *p*-value and the number of genes in the corresponding term, respectively. LN, lupus nephritis; GO, gene ontology.

From the heatmap of module-trait relationships, we found that multiple modules were associated with LN (Figure 3A). Then, we calculated the gene significance of all genes in 12 co-expression modules (Figure 3B). The results showed that the darkgreen module was most significantly related to LN, followed by the purple module. The correlation between MM and GS in these two modules are shown in Figure 3C, which mean that MM was highly correlated with GS for LN in both darkgreen module and purple module (cor = 0.77, *p* = 5.8e−76, and cor = 0.41, *p* = 5.2e−15, respectively).

To investigate the potential biological function of genes in the above two modules, we performed GO enrichment analysis. The top 20 significant GO biological process terms of each module were shown in Figures 3D,E. We found that genes in the purple module were mainly enriched in defense response to virus, type I interferon signaling pathway, immune response, and inflammatory response, which are highly associated with LN. However, genes in the darkgreen module were mostly distributed in terms like cell diversion and proliferation, mitotic nuclear division, and DNA replication, which mainly play roles in metabolism. Thus, we defined the purple module as the key module related to LN and performed further analysis.

### Identification of Hub Genes

Hub genes should be DEGs between LN patients and normal controls. In dataset GSE99967, a total of 137 DEGs were identified, including 84 up-regulated genes and 53 down-regulated genes (Supplementary Table 2). The heatmap and volcano plot of all DEGs are shown in Supplementary Figures 2, 3.

To identify hub genes in the key module, we calculated MCC values of genes in the purple module and extracted the top 10 genes with higher scores (Supplementary Figure 4). Among these ten genes, 5 DEGs were identified as hub genes, including interferon induced protein 44 (IFI44), interferon induced protein with tetratricopeptide repeats three (IFIT3), HECT and RLD domain containing E3 ubiquitin protein ligase 5 (HERC5), radical S-adenosyl methionine domain containing two (RSAD2), and DExD/H-box helicase 60 (DDX60). The GS and MM value of these genes were all > 0.4 and 0.9, respectively, indicating that hub genes were significantly associated with the key module and LN trait.

### Validation of Hub Genes by Other Datasets and qRT-PCR

**Figure 4:**
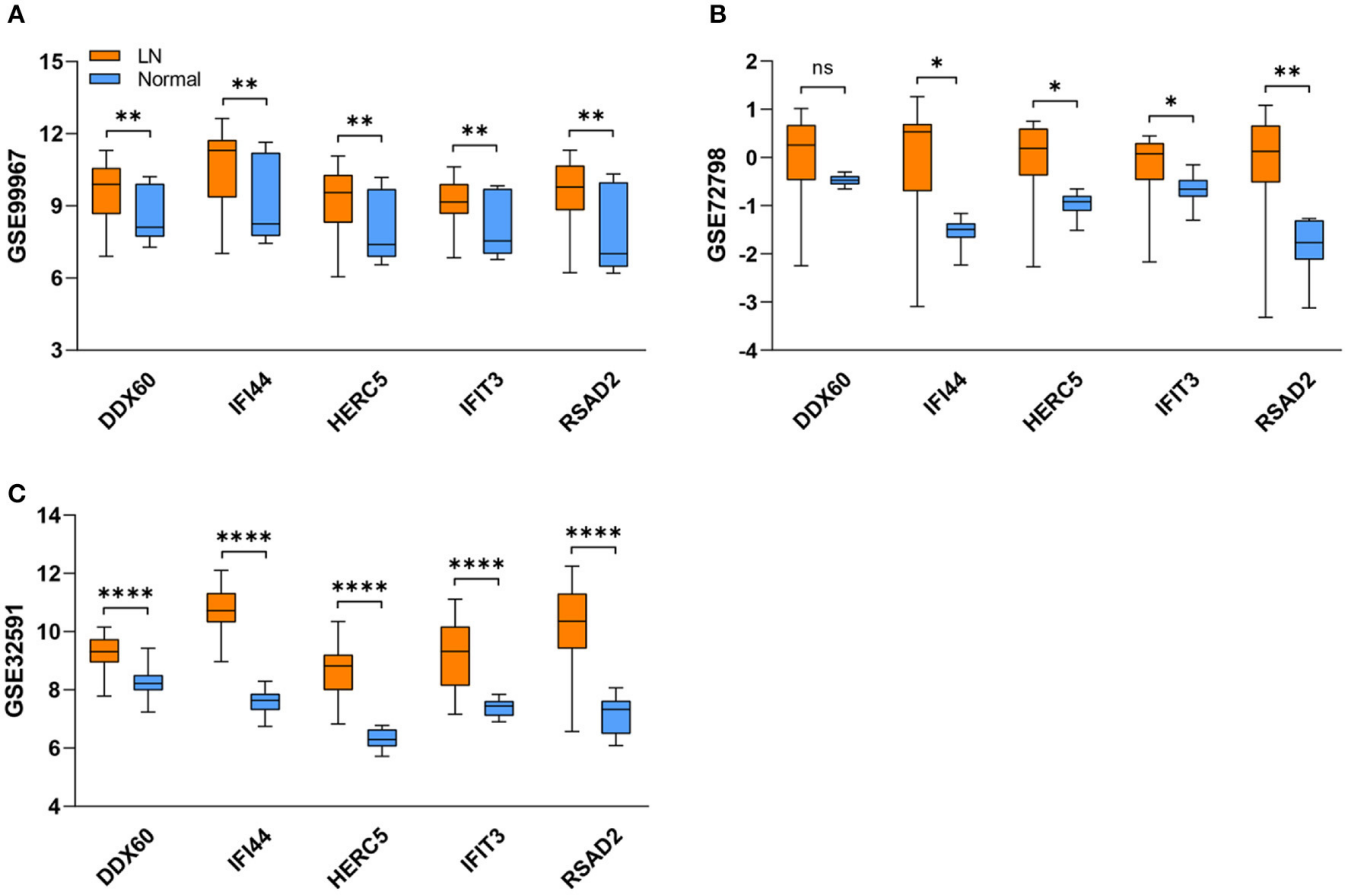
Validation of five hub genes in datasets. **(A–C)** The blue box refers to the normal group and the orange one refers to the LN group. Box represents mean ± SD by an unpaired *t*-test. *P < 0.05, **P < 0.01, ***P < 0.0001, ns, no significance; LN, lupus nephritis.

As expected, the expression levels of hub genes including IFI44, IFIT3, HERC5, RSAD2, and DDX60 were significantly upregulated in LN samples from the GSE99967 dataset (Figure 4A). For verifying hub genes, we obtained another two datasets GSE72798 and GSE32591, and analyzed the expression levels of the above five genes between LN patients and normal controls (Figures 4B,C). Except for DDX60 in GSE72798, the other four hub genes were markedly increased in LN samples.

In addition, to further validate these four hub genes, we collected nine blood samples from four healthy controls and five LN patients (patient characteristics are shown in Supplementary Table 3) to perform qRT-PCR. The results showed that compared with HCs, the mRNA levels of IFI44, IFIT3, HERC5, and RSAD2 were all significantly elevated in LN patients (Supplementary Figure 5), meaning that they are expected to be potential biomarkers in identifying LN.

### ELISA Validation

**Figure 5:**
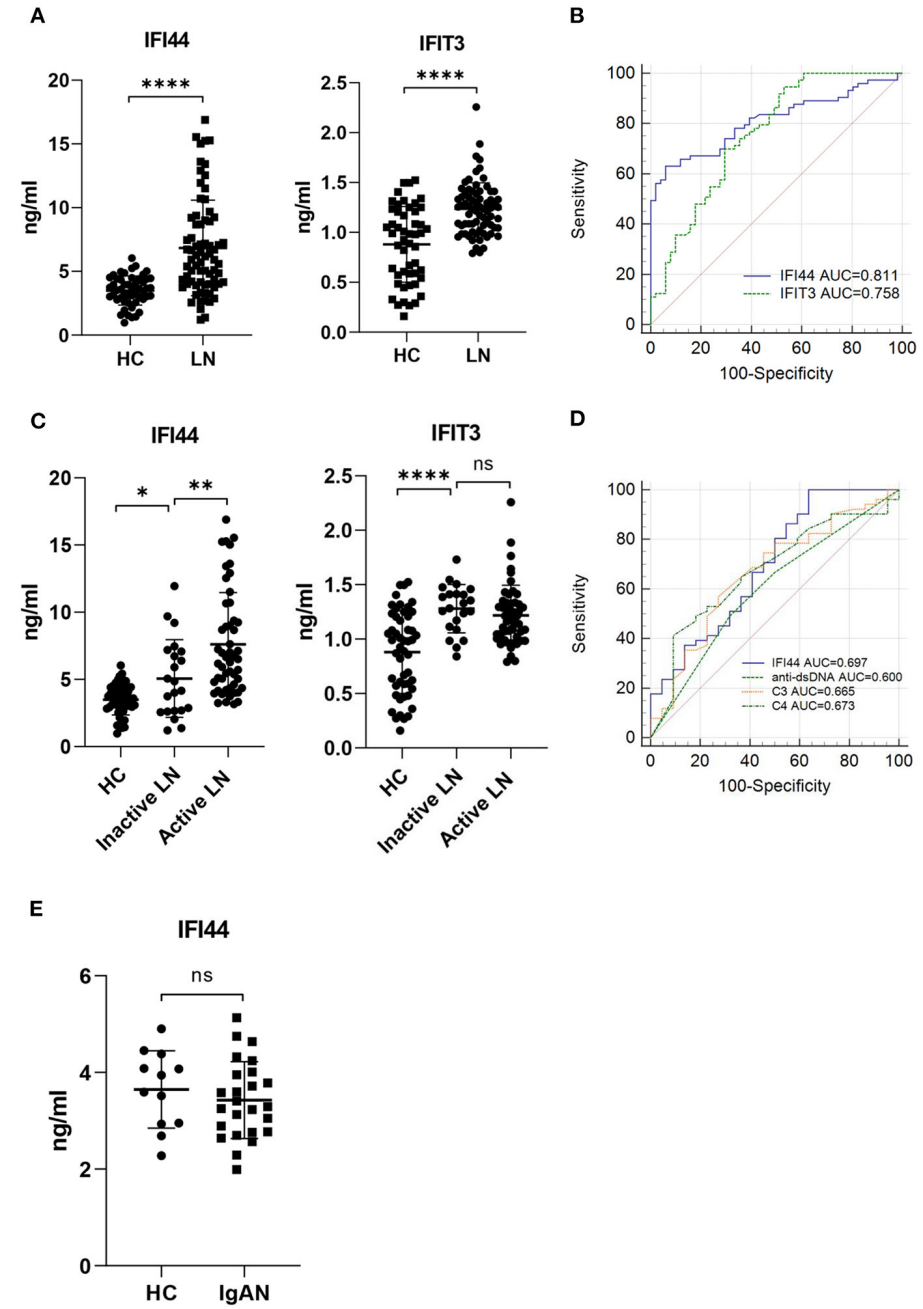
ELISA validation and ROC analysis. **(A)** Serum IFI44 (Left) and IFIT3 (Right) were measured in 51 healthy controls and 73 LN patients by ELISA. **(B)** Receiver operator characteristic (ROC) curves show the diagnostic performance of serum IFI44 (blue) and IFIT3 (green) in identifying LN patients. **(C)** 73 LN patients were divided into two groups (Inactive LN=22, Active LN=51) according to renal biopsy and the Systemic Lupus Erythematosus Disease Activity Index (SLEDAI). **(D)** ROC curves of IFI44, anti-dsDNA, C3, and C4 in differentiating active LN from inactive LN patients. **(E)** Serum IFI44 was tested in 12 healthy controls and 25 patients with IgA nephropathy. Data shown are mean ± SD by an unpaired *t*-test. *P < 0.05, **P < 0.01, ****P < 0.0001, ns, no significance; HC, healthy control; LN, lupus nephritis; IgAN, immunoglobulin A nephropathy.

Among five hub genes, we selected type I interferon-response genes IFI44 and IFIT3 for further analysis. To detect the serum levels of IFI44 and IFIT3, 124 subjects, including 51 healthy controls and 73 LN patients (patient characteristics are shown in Supplementary Table 4) were used to perform ELISA assays (Figure 5). As expected, IFI44 and IFIT3 were markedly upregulated in LN patients compared with the healthy controls (Figure 5A). Furthermore, ROC curves were used to calculate the sensitivity and specificity of these two biomarkers for identifying LN patients. The AUC values of IFI44 and IFIT3 were 0.811 and 0.758, respectively (Figure 5B, Supplementary Table 5). Then, we combined these two biomarkers using logistic regression analysis (Supplementary Figure 6A, Supplementary Table 6). However, the AUC value of combined score (AUC = 0.811) was the same as IFI44 (Supplementary Figure 6B), indicating that IFI44 had the highest power to distinguish between the two groups. Additionally, we found that IFI44 was significantly elevated in active LN compared with inactive LN patients, while IFIT3 failed to discriminate active LN from inactive ones (Figure 5C). And the AUC of IFI44 in differentiating active LN from inactive LN patients was 0.697, which was similar to clinical indicators such as anti-dsDNA (AUC = 0.600), C3 (AUC = 0.665), and C4 (AUC = 0.673) (Figure 5D; Supplementary Table 7).

To examine whether serum IFI44 and IFIT3 can reflect the pathological class of kidney biopsy, we classified 73 LN patients into five groups according to LN class. The results showed that they were both elevated in class III(±V), class IV(±V), and class V compared with HCs (Supplementary Figure 7), meaning that both IFI44 and IFIT3 can indicate the prognosis of LN.

Moreover, we identified the specificity of IFI44 for LN. An independent cohort of 37 subjects, including 12 healthy controls and 25 patients with IgA nephropathy (patient characteristics are shown in Supplementary Table 8), were used for investigation. Interestingly, there was no statistical difference between HCs and patients with IgA nephropathy (Figure 5E), indicating that IFI44 is specific for LN.

## Discussion

Lupus nephritis is a major cause of morbidity and mortality in SLE, and many patients end in chronic kidney disease (CKD) or ESRD due to limited drug treatment (17, 18). Therefore, it is imperative to develop candidate biomarkers and potential therapeutic targets to improve the prognosis of patients with LN. In this study, we used the GSE99967 dataset to screen the hub genes associated with the pathogenesis of LN. WGCNA was utilized to construct co-expression modules associated with LN, and GO enrichment analysis was applied to explore the biological function of genes in the key module. Five hub genes were obtained using the MCC algorithm, including IFI44, IFIT3, HERC5, RSAD2, and DDX60. qRT-PCR and ELISA assays were performed to validate potential biomarkers in independent cohorts. We found that both IFI44 and IFIT3 have diagnostic accuracy in identifying LN, especially IFI44, which is specific for LN. Our findings may shed light on the pathogenetic basis of LN and provide candidate biomarkers for its treatment.

We found that genes in the key module were mainly enriched in defense response to virus and type I interferon signaling pathway, which are consistent with previous studies (19, 20). Anders HJ et al. indicated that endogenous RNA-related autoantigens can be recognized by Toll-like receptor-7 (TLR7), a viral nucleic acid recognition receptor that triggers antiviral immunity, thus aggravating lupus nephritis (21). In contrast, blockade of TLR7, TLR9, or both attenuates lupus nephritis (22). Additionally, antiviral genes such as ISG15, MX1, and the OAS gene family are involved in the pathogenesis of LN (23). Likewise, type I interferon signaling pathway-related genes, such as IRF5 and STAT4, are associated with the risk of LN (24). Triantafyllopoulou A et al. revealed the role of intrarenal type I IFNs in kidney damage of LN (25). Furthermore, monitoring the peripheral blood type I interferon signature can track the kidney disease activity in patients with SLE (26). Therefore, hub genes we obtained from the key module were confirmed to be highly relevant to LN.

Both IFI44 and IFIT3 are type I IFN signature genes, which may contribute to the pathogenesis of autoimmune diseases (27, 28). In independent cohorts, we found that serum IFI44 and IFIT3 can discriminate LN patients from healthy controls, which may act as candidate biomarkers in identifying LN. Similar to our findings, increased gene expression of IFI44 and IFIT3 was observed in SLE patients compared to HCs (29). What's more, IFI44, as an LN-specific biomarker, can distinguish between active LN patients and inactive ones. Previous studies have shown that patients with class III, IV, or V LN, but not class I or II LN, are at direct risk of CKD progression. What's worse, both class III and class IV LN involve irreversible nephron loss, which shortens kidney lifespan (30). Interestingly, our results revealed that IFI44 and IFIT3 were both elevated in class III(±V), class IV(±V), and class V LN, suggesting that these two biomarkers can indicate the prognosis of LN. Also, IFIT3 may proposed as a novel therapeutic target for blocking the production of type I IFN in patients with SLE (31). In terms of epigenetics, IFI44 and IFIT3 were hypomethylated in comparisons between lupus patients and non-lupus subjects (32, 33). Moreover, both IFI44 and IFIT3 have antiviral activity, meaning that they may be involved in the anti-endogenous nucleic acid response of LN patients (34, 35).

In addition, HERC5, RSAD2, and DDX60 are all interferon induced-genes, which play important roles in antiviral response (36–39). Consistent with our findings, recent studies revealed that the expression level of RSAD2 was markedly increased in the SLE samples compared with controls, which may act as a potential biomarker gene and therapeutic target for the treatment of SLE (40, 41). Similarly, upregulated gene expression of DDX60 was observed in the IFN-I-positive childhood-onset SLE patients (42). Due to the epigenetic susceptibility of lupus, Coit P et al. found that HERC5 is hypomethylated in lupus patients with renal involvement (43). Thus, it is important to clarify the relationship between these five hub genes and the pathogenesis of LN.

Our findings provide new insights into the occurrence, progression of LN patients. Sun G et al. explored the genes genetically associated with LN through bioinformatics analysis (44). They found that LN-related genes were mainly involved in immune and inflammatory responses, which are in agreement with what we found. Likewise, the GSE104948 dataset was analyzed by WGCNA, and CD36 was ultimately screened out as a hub gene of the pathogenesis of LN (45). However, neither of their findings have been validated in LN patients. Moreover, Yao M et al. used dataset GSE32591 to explore the pathophysiological changes in glomeruli and tubulointerstitia of LN patients (46). Consistent with our findings, they found that type I interferon response was highly active in LN and the crosstalk genes, such as IRF7, HLA-DRA, ISG15, PSMB8, and IFITM3 were validated in six samples. However, in our study, after identifying the hub genes, we verified the mRNA expression and serum concentration of them with large sample size, and they are expected to be candidate biomarkers and potential therapeutic targets of LN.

However, our study contains some limitations. First, patients in validation cohorts are composed of Chinese. Hence, our findings may not generalize LN patients from different races and ethnicities. Second, samples for qRT-PCR validation are limited. Thus, more samples are needed to verify the mRNA expression of these hub genes. Third, the exact mechanisms of the identified hub genes in the occurrence and progression of LN need to be further investigated.

In conclusion, based on WGCNA, we identified five hub genes, including IFI44, IFIT3, HERC5, RSAD2, and DDX60, which are highly correlated with LN. GO enrichment analysis revealed that these genes are mainly enriched in defense response to virus, type I interferon signaling pathway and immune response. In validation cohorts, IFI44 was found to be a candidate biomarker to diagnose diseases or assess prognosis. Our findings provide potential therapeutic targets and shed light on the pathogenetic basis of LN.

## Data Availability Statement

The raw data supporting the conclusions of this article will be made available by the authors, without undue reservation.

## Ethics Statement

The studies involving human participants were reviewed and approved by the Research Ethics Committee of the First Affiliated Hospital, College of Medicine, Zhejiang University. The patients/participants provided their written informed consent to participate in this study.

## Author Contributions

JC, HJ, and LS designed the study. LS and TZ conducted bioinformatics. LS, LL, HC, HG, and PE carried out experiments. YC, MW, and HT performed the statistical analysis. LL and CW wrote the manuscript. All authors contributed to the manuscript and approved the submitted version.

## Funding

This work was supported by Sino-German center (M-0144).

## Conflict of Interest

The authors declare that the research was conducted in the absence of any commercial or financial relationships that could be construed as a potential conflict of interest. The reviewer JM declared a shared affiliation with the authors to the handling editor at time of review.

## Publisher's Note

All claims expressed in this article are solely those of the authors and do not necessarily represent those of their affiliated organizations, or those of the publisher, the editors and the reviewers. Any product that may be evaluated in this article, or claim that may be made by its manufacturer, is not guaranteed or endorsed by the publisher.
