# Supplementary material for: Identification and Validation of IFI44 as Key Biomarker in Lupus Nephritis
Source: Front Med (Lausanne). 2021 Oct 25;8:762848. doi: 10.3389/fmed.2021.762848 (PMC8574154; doi:10.3389/fmed.2021.762848)
Supplement: Supplementary file 1 [file Data_Sheet_1.PDF]

Supplementary Figures

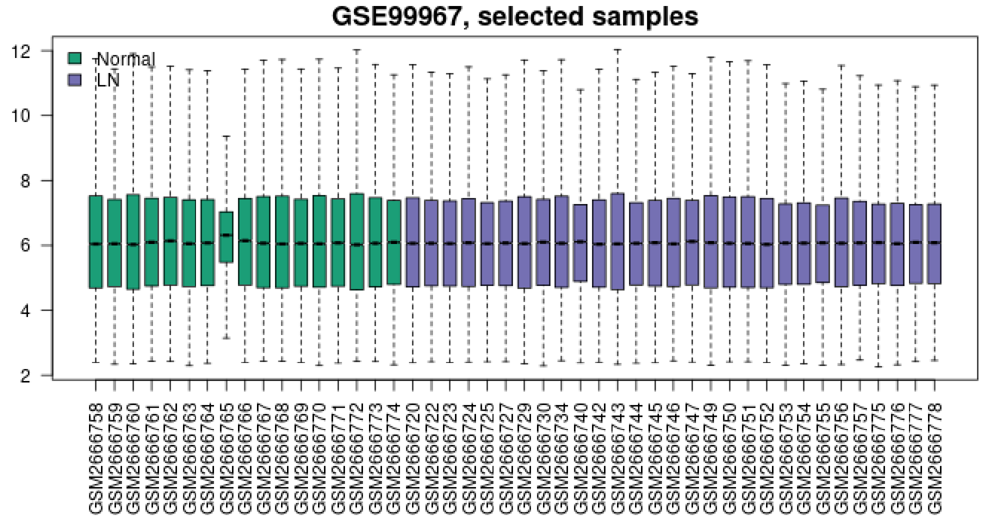

Supplementary Figure 1. Cross comparison of two groups of LN patients and normal controls. The green boxes refer to normal controls and the blue ones refer to LN patients.

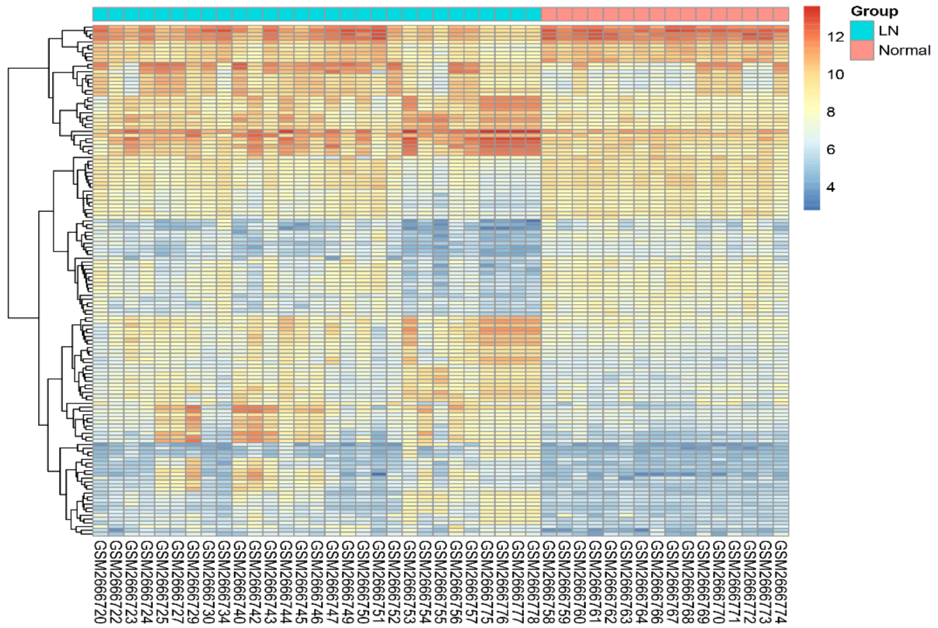

Supplementary Figure 2. Heatmap of all differentially expressed genes.

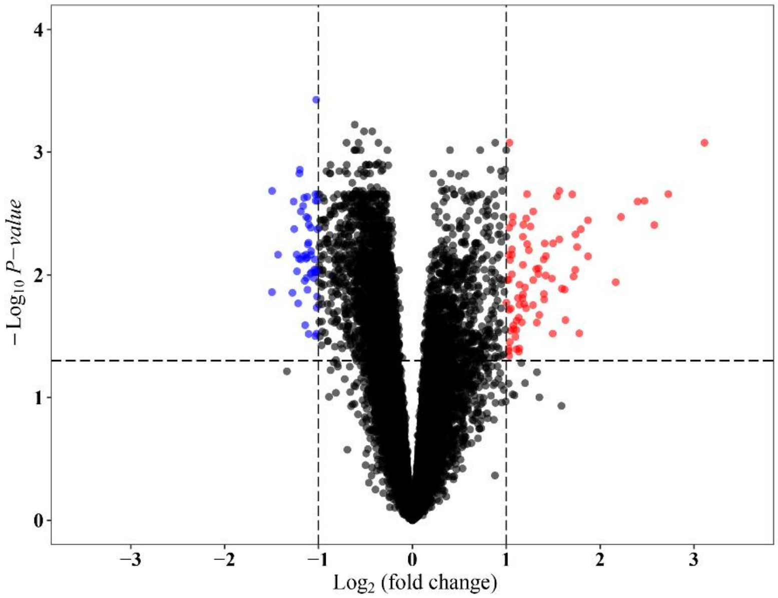

Supplementary Figure 3. Volcano plot of all differentially expressed genes.

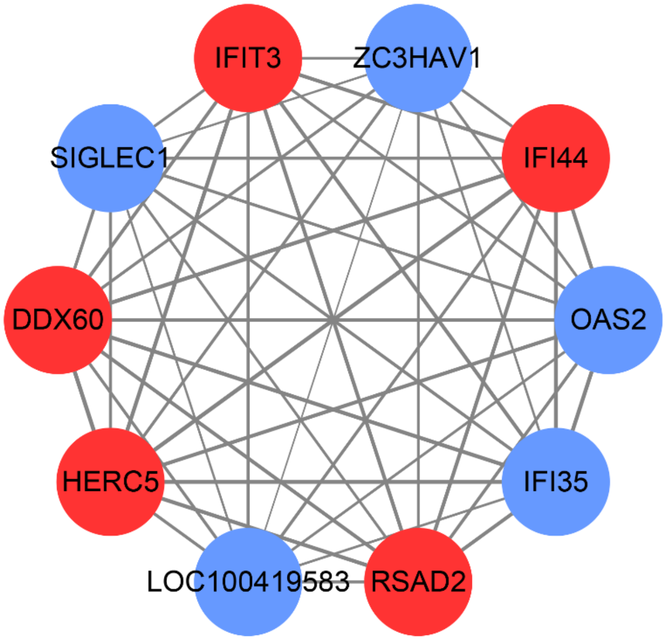

Supplementary Figure 4. Top 10 genes with higher MCC values. The nodes represent the genes (red for differentially expressed genes) and the edges represent the weighted correlation.

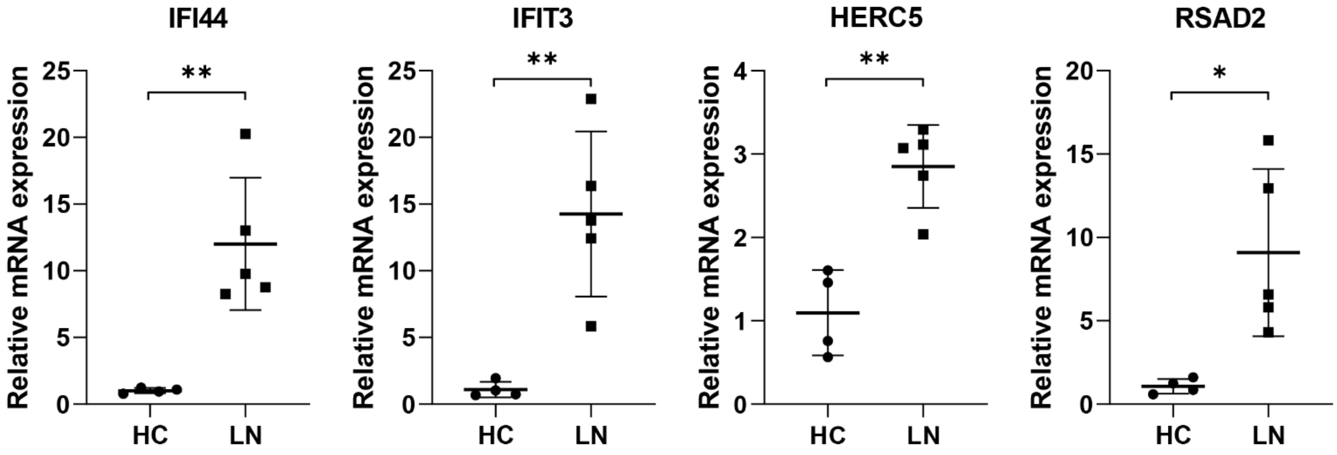

Supplementary Figure 5. qRT-PCR validation. Relative mRNA expression of IFI44, IFIT3, HERC5, and RSAD2 were measured in 4 healthy controls and 5 LN patients. Data shown are mean ± SD by an unpaired t-test; *P < 0.05, **P < 0.01. HC, healthy control; LN, lupus nephritis.

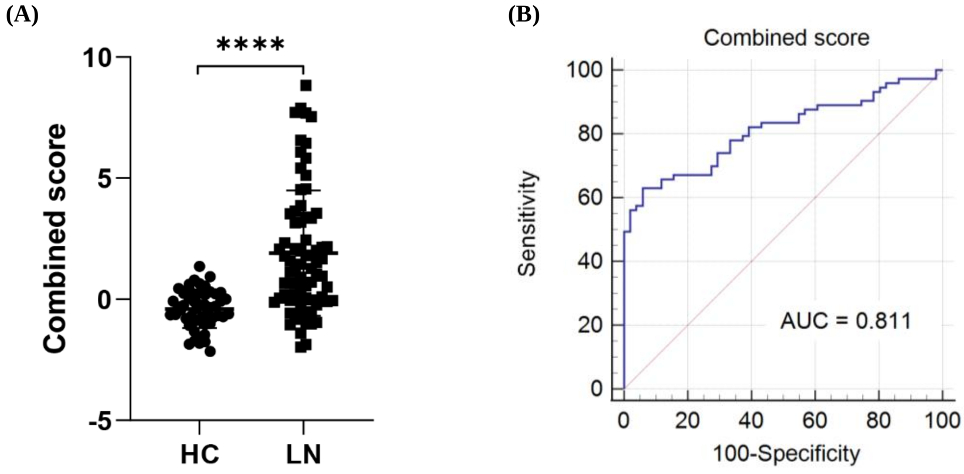

Supplementary Figure 6. Combination of IFI44 and IFIT3. (A) Combined score in HC and LN. (B) The receiver operating characteristic (ROC) curve shows the diagnostic performance of the combined score in identifying LN patients. Data shown are mean ± SD by an unpaired t-test; ****P < 0.0001. HC, healthy control; LN, lupus nephritis.

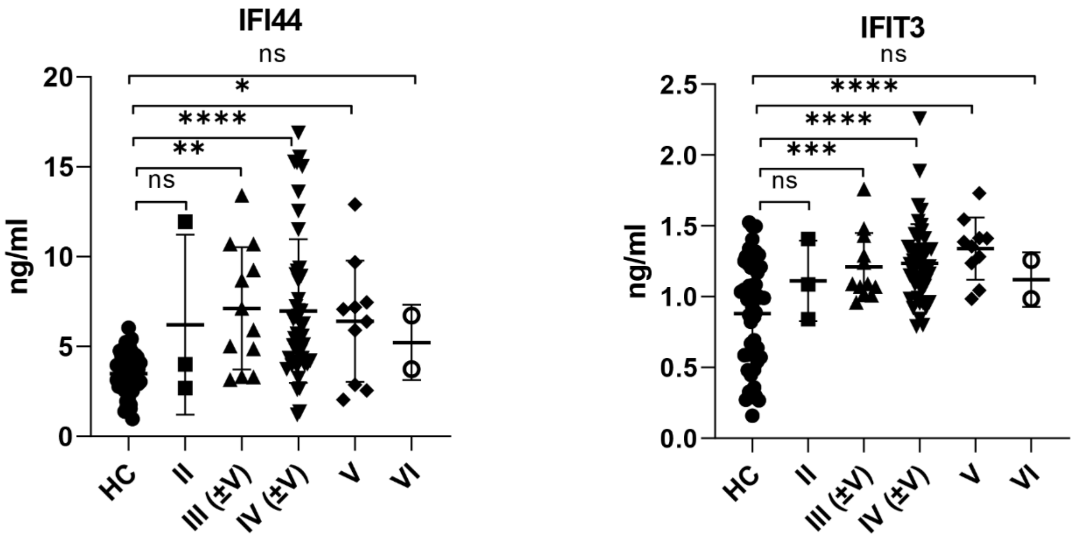

Supplementary Figure 7. The serum levels of IFI44 and IFIT3 in LN patients with different LN class. 73 LN patients were divided into five groups (class II=3, class III(±V)=12, class IV(±V)=46, class V=10, class VI=2) according to LN class. Data shown are mean ± SD by an unpaired t-test; *P < 0.05, **P < 0.01, ***P < 0.001, ****P < 0.0001, ns, no significance. HC, healthy control; LN, lupus nephritis.
