# Supplementary material for: Identification and Validation of IFI44 as Key Biomarker in Lupus Nephritis
Source: Front Med (Lausanne). 2021 Oct 25;8:762848. doi: 10.3389/fmed.2021.762848 (PMC8574154; doi:10.3389/fmed.2021.762848)
Supplement: Supplementary file 2 [file Data_Sheet_2.PDF]

Supplementary Tables

Supplementary Table 1. All primer sequences used in this study.

Primers	Forward (5' -3')	Reverse (5'-3')
IFI44	TCCAAGGGCATGTAACGCAT	CCTCCCTTAGATTCCCTATTTGCT
IFIT3	GAAACAGCCATCATGAGTGAGG	GCATCTGAGAGTCTGCCCAA
HERC5	AACCTGCATGGGCAGCTTGG	TGTGGGCTTCTCCGGCAGAA
RSAD2	CGTGGAAGAGGACATGACGG	GCATCTGAGAGTCTGCCCAA
GAPDH	GGCATCCTGGGCTACACTGA	GGAGTGGGTGTCGCTGTTG

Supplementary Table 3. Characteristics and medications of patients for qRT-PCR validation.

	HCs	LN
n	4	5
Age (years)	35.3±11.5	38±10.8
Female(%)	75.0%	80.0%
SLEDAI	-	10.4±4.2
Anti-dsDNA(%)	-	60.0%
C3 (mg/dl）	-	62.0±28.3
C4 (mg/dl）	-	8.0±4.3
Medication(%)	-	Pred(80%), MMF(40%), HCQ(40%), Cyc(20%), CyA(20%)

Pred, prednisone; MMF, mycophenolate mofetil; HCQ, hydrochloroquine; Cyc, cyclophosphamide; CyA, Ciclosporin A; HC, healthy control; LN, lupus nephritis; SLEDAI, Systemic Lupus Erythematosus Disease Activity Index.

Supplementary Table 4. Characteristics and medications of patients for ELISA validation.

	**HCs**	**All LN**	**Active LN**	**Inactive LN**
n	51	73	51	22
Age (years)	34.4±7.9	34.2±12.3	33.7±12.3	35.3±12.3
Female(%)	82.4%	83.6%	80.4%	90.9%
SLEDAI	-	13.4±7.2	17.3±3.5	4.2±2.2
Anti-dsDNA(%)	-	61.6%	64.7%	54.5%
C3 (mg/dl）	-	48.3±24.0	44.4±22.1	57.5±26.2
C4 (mg/dl）	-	8.5±7.1	7.7±7.2	10.5±6.6
Medications(%)	-	Pred(95.9%), MMF(42.5%), TAC(30.1%), Cyc(8.2%), Aza(6.8%)	Pred(98.0%), MMF(49.0%), TAC(27.5%), Cyc(7.8%), Aza(9.8%)	Pred(90.9%), MMF(27.3%), TAC(36.4%), Cyc(9.1%), Aza(0%)

Pred, prednisone; MMF, mycophenolate mofetil; TAC, tacrolimus; Cyc, cyclophosphamide; Aza, azathioprine; HC, healthy control; LN, lupus nephritis; SLEDAI, Systemic Lupus Erythematosus Disease Activity Index.

Supplementary Table 5. The diagnostic performance of serum IFI44 and IFIT3 in identifying LN patients.

Variable	AUC	SE	95% CI
IFI44	0.811	0.0382	0.731 to 0.876
IFIT3	0.758	0.0453	0.673 to 0.830

Pairwise comparison of ROC curves

**IFI44 ~ IFIT3**	
Difference between areas	0.0526
Standard Error	0.0285
95% Confidence Interval	-0.00314 to 0.108
z statistic	1.85
Significance level	P = 0.0643

Supplementary Table 6. Logistic regression analysis of the serum level of IFI44 and IFIT3.

	B	SE	WALD	df	Sig.	Exp	95%CI
IFI44	0.0689	0.262	6.887	1	0.009	1.991	1.190-3.330
IFIT3	0.006	1.352	0.000	1	0.997	1.006	0.071-14.220
Constant	-2.819	0.821	11.779	1	0.001	0.060	-

Combined score=-2.819+0.0689*IFI44+0.006*IFIT3. B, regression coefficient; SE, Standard Error; WALD, WALD Chi-square; df, degree of freedom; Sig., Significance; Exp(B), Odds ratio; CI, confidence interval.

Supplementary Table 7. Serum IFI44 and clinical indicators in differentiating active LN from inactive LN patients.

**Variable**	**AUC**	**SE**	**95% CI**
IFI44	0.697	0.0709	0.578 to 0.799
Anti-dsDNA	0.6	0.0678	0.478 to 0.713
C3	0.665	0.0703	0.545 to 0.772
C4	0.673	0.0681	0.554 to 0.779

Pairwise comparison of ROC curves

**IFI44 ~ Anti-dsDNA**	
Difference between areas	0.0971
Standard Error	0.1
95% Confidence Interval	-0.0997 to 0.294
z statistic	0.967
Significance level	P = 0.3334

**IFI44 ~ C3**	
Difference between areas	0.0316
Standard Error	0.0877
95% Confidence Interval	-0.140 to 0.203
z statistic	0.361
Significance level	P = 0.7182

**IFI44 ~ C4**	
Difference between areas	0.0236
Standard Error	0.0869
95% Confidence Interval	-0.147 to 0.194
z statistic	0.272
Significance level	P = 0.7858

Supplementary Table 8. Characteristics of patients with IgA nephropathy.

	**HCs**	**IgAN**
n	12	25
Age (years)	34±5.9	33.7±8.1
Female(%)	83.3%	88.0%
Renal biopsy	-	
M0/M1(n)		22/3
E0/E1(n)		25/0
S0/S1(n）		7/18
T0/T1(n）		23/2
C0/C1(n）		16/9
